# Multiple Myeloma as the Underlying Cause of Thrombotic Microangiopathy Leading to Acute Kidney Injury: Revisiting a Very Rare Entity

**DOI:** 10.1177/2324709617732797

**Published:** 2017-09-22

**Authors:** Savneek Chugh, Asim Kichloo, Firas Jafri, Liga Yusvirazi, Robert Lerner

**Affiliations:** 1 New York Medical College, Valhalla, NY, USA; 2 Central Michigan University, St Mary’s Hospital, Saginaw, MI, USA

**Keywords:** thrombotic microangiopathy, microangiopathic hemolytic anemia, thrombotic thrombocytopenic purpura, hemolytic uremic syndrome, multiple myeloma

## Abstract

Thrombotic microangiopathy (TMA) describes a pathological process of microvascular thrombosis, consumptive thrombocytopenia, and microangiopathic hemolytic anemia, leading to end-organ ischemia and infarction, affecting particularly the kidney and brain. TMA is a pathological feature of a number of clinical disorders including thrombotic thrombocytopenic purpura, hemolytic uremic syndrome, and atypical hemolytic uremic syndrome. Rare but important, TMA may also occur in malignancy, connective tissue disease, malignant hypertension, and renal transplantation (rejection or drug toxicity). We present a very rare case where the patient developed acute kidney injury from TMA but found to have multiple myeloma as the possible underlying etiology.

## Introduction

Thrombotic microangiopathy (TMA) is a pathologic pattern of injury, characterized by systemic microthrombi, hemolytic anemia, and thrombocytopenia, leading to end-organ ischemia affecting particularly the kidney and brain.^1^ The pathologic feature of TMA syndromes is arteriolar and capillary thrombosis causing vascular damage with characteristic abnormalities in the endothelium and vessel wall.^1,2^

The new classification of TMA proposed by Dr James George classifies TMA under primary and secondary disorders.^1^ Primary TMA is composed of 9 disorders, and the most commonly discussed are thrombocytopenic thrombotic purpura and hemolytic uremic syndrome. Secondary TMAs are related to malignancy, connective tissue disease, malignant hypertension, pregnancy, and renal or stem cell transplantation (rejection or drug toxicity).^1^ Plasma cell dyscrasias are frequently encountered malignancies often associated with kidney disease through the production of immunoglobulin.^2^ In this article, we are present and discuss a very rare case of TMA, which was later diagnosed to have an underlying multiple myeloma (MM).

## Case Report

A 42-year-old Haitian man with past medical history of mixed connective tissue disease with pulmonary hypertension on home oxygen and chronic diastolic heart failure with preserved ejection fraction presented to an outside hospital with shortness of breath, weight loss, fatigue, and pedal edema for about 14 days. He was diagnosed with rapidly progressing acute kidney injury (AKI) and was transferred to our tertiary care hospital for further management. During the hospitalization, he was found to have a serum creatinine of 3.07 mg/dL and urinalysis showing 2+ proteins and 2+ blood with dysmorphic red blood cells on microscopy. At admission hemoglobin was 6.0 g/dL, mean corpuscular volume 87.5 fL, hematocrit 20.3%, white blood cell count was 5300/m^3^, neutrophils were 90.0%, and platelet count of 130 000/mm^3^. Viral serologies of hepatitis B, hepatitis C, and HIV were negative. Quantiferon TB Gold test for tuberculosis was negative. The serological tests for autoimmune diseases, which include ANA, ANCA, HLA-B27, anti-Smith antibody, anti SS-A, anti SS-B, anti-centromere B antibody, anti-Jo antibody, myeloperoxidase antibody, and cyclic citrullinated peptide, were negative except for anti-RNP and ESR. Serum albumin was 2.1 g/dL, and serum globulin was 4.7 g/dL.

**Figure 1.**
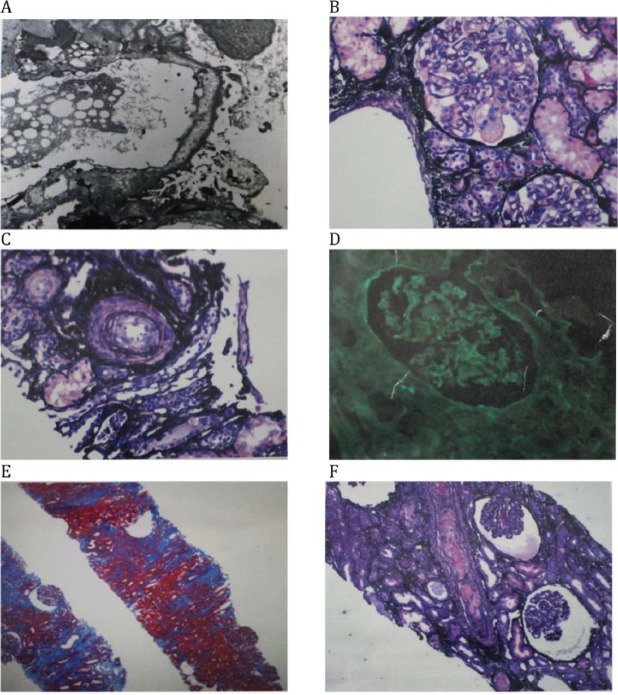
Kidney biopsy. (A) Electron microscopy. No immune-type electron dense deposits in mesangial or capillaries walls. There is mild effacement of podocyte foot processes. (B) Jones; mesangiolysis. (C) Jones; interstitial edema. (D) Direct immunofluorescence. No significant glomerular and tubular staining. (E) Trichrome; focal interstitial fibrosis. (F) Light microscopy. Jones; fibrin thrombus in artery and ischemic glomeruli.

Percutaneous renal biopsy under light microscopy showed some glomeruli with ischemic changes characterized by wrinkling glomerular capillary walls. Mild interstitial fibrosis and tubular atrophy was seen and a few arteries and arterioles showed fibrin thrombi in the vessel lumen. There was mild to moderate arteriosclerosis. Under direct immunofluorescence there was no significant glomerular staining for IgG, IgA, IgM, C3, and C1q, fibrinogen, albumin, and kappa and lambda light chains. There was nonspecific tubular cast staining for IgA and kappa and lambda light chains. There were no immune-type electron dense deposits in mesangial regions, tubular basement membranes, interstitium, peritubular capillaries, or capillary walls by electron microscopy; however, mild effacement of podocyte foot processes was seen. The biopsy was interpreted as suggestive of renal TMA with mild tubular atrophy and interstitial fibrosis (see Figure 1).

The patient remained anemic with hemoglobin of 8 g/dL requiring 3 red blood cell transfusions over the hospitalization of 4 weeks. Additional laboratory results of lactate dehydrogenase 359 U/L, haptoglobin <8 mg/dL, and schistocytes in the peripheral blood smear suggested hemolysis. A platelet count of 74 000/mm^3^ with normal megakaryocytes in the bone marrow done later suggested consumptive thrombocytopenia. With the evidence of ongoing hemolysis, thrombocytopenia, and renal failure, a diagnosis of TMA was made and the patient was started on plasma exchange of 1.5 plasma volumes daily for 5 days. Despite the treatment renal function worsened and hemodialysis was initiated.

After 25 days in hospital, urine volume increased and creatinine stabilized around 2.0 mg/dL. Workup of TMA prior to plasma exchange showed normal complement levels with C3 level of 119.0 mg/dL, complement C4 level of 36.70 mg/dL, and ADAMTS 13 activity level of 57%. Serum protein electrophoresis showed albumin 32.66% of a total protein 6.40 g/dL and M-spike 30.05%. Serum immunofixation identified the M-spike as IgG at a level of 2199 mg/dL. Serum kappa free light chain was 18.2 mg/dL, lambda free light chain was 5.28 mg/dL, and an elevated kappa/lambda ratio of 3.45. Urine protein was 1994 mg/g of creatinine, which was 15.1% globulin and 3.7% monoclonal by electrophoresis. A bone marrow biopsy showed clonal plasmacytosis consistent with plasma cell dyscrasia with 18% plasma cells. Unfortunately, the patient developed severe sepsis and died before initiation of management for MM.

## Discussion

MM accounts for 1% of neoplastic disease and 13% of hematological malignancies. Renal impairment occurs in 20% to 40% of patients with MM and more than 80% have proteinuria.^3^ The mechanisms of kidney injury can be grouped into immunoglobulin-dependent and immunoglobulin-independent categories. The 3 most common forms of monoclonal immunoglobulin-mediated kidney disease are cast nephropathy, monoclonal immunoglobulin deposition disease, and AL amyloidosis. The other rare causes of kidney injury in MM are glomerulonephritis, tubulointerstitial nephritis, minimal change disease, IgA nephropathy, and hyperviscosity syndrome.^4^ Myelomas that produce only light chains are responsible for 40% to 60% of severe myeloma-associated kidney injuries, reflecting the nephrotoxicity of the filtered light chains.^4^

Our case was unique as TMA was the initial presentation, which is very rare for kidney injury induced by MM. In this case kidney failure developed acutely and required renal replacement therapy. Laboratory workup was consistent with microangiopathic hemolysis with normal ADAMTS13 activity level and normal complement levels. Kidney biopsy showed no immune deposit but there were microthrombi in a few arteries and arterioles. Serum and urine electrophoresis, serum immunofixation, and bone marrow biopsy result confirmed the diagnosis of MM. The patient developed sepsis and died before definitive treatment for MM was started.

Various mechanisms of TMA in MM have been proposed.^4^ TMA in MM patients can result from either direct Ig-induced endothelial injury, or IgG antibody against ADAMTS13 leading to thrombocytopenic thrombotic purpura. The pathogenesis of Ig-induced endothelial injury involves the light chains that are filtered through the glomerulus. They undergo chemical degradation and induce proinflammatory cytokines, such as IL-6 and IL-8. These light chains lead to formation of redox-sensitive transcription factor NF-κB and mitogen-activated protein kinase, which in turn results in inflammatory cell infiltration, matrix deposition, and fibrosis.^5^

Other suggested mechanisms of TMA are that systemic chemotherapy or stem cell transplantation damage endothelium.^6^ There is one case report of the successful use of eculizumab in a patient with MM and TMA suggesting a complement-mediated mechanism that has not been investigated.^7^

In one autopsy series of 77 MM patients there were 10 cases of TMA, some after allogenic hematopoietic stem cell transplant.^6^ Another article reported 9 cases of TMA with MM. Of these, 4 patients had TMA as the initial presentation of MM, and 5 patients were on treatment, most often bortezomib-based regimens. Another study demonstrates that TMAs can occur in MM outside of the context of allogenic hematopoietic stem cell transplant. In roughly one third of patients, TMAs were the initial presentation of MM and improved with treatment.^8^ There are reported cases suggesting that an acquired von Willebrand factor disease might develop during the course of MM.^7,9-11^ In these patients, the suggested mechanism is the prevention of the interaction of GP1b with von Willebrand factor due to the paraproteins.^7,9-12^ Another study reported renal function improvement after administration of a monoclonal antibody against C5 (eculizumab) in a post–bone marrow transplant MM patient.^13^ This leads to the hypothesis that complement or complement-mediated factors can also be one of the causative factors of renal damage in MM patients. Little data are available to support this statement.

Signs and symptoms of myeloma and plasma cell dyscrasias are nonspecific and include weight loss, malaise, fatigue, bone pain, anemia, and thrombocytopenia. Similarly, the symptoms of the kidney injury are also nonspecific, and occasionally the patient will present with a fulminant syndrome of dialysis-dependent AKI; thus, primary care physicians, nephrologists, and hematologists/oncologists should consider plasma cell dyscrasias in the differential diagnosis of AKI and hematological abnormalities including TMA.

The deadly consequences of TMA in MM as seen in our patient warrant further studies to be done in order to learn more about the pathophysiology including possible role of ADAMTS13, complement, and complement-mediated factors. Further studies are also required to define the potential benefit of treatment with plasmapheresis for TMA in MM.
